# Modeling the genetic relatedness of *Plasmodium falciparum* parasites following meiotic recombination and cotransmission

**DOI:** 10.1371/journal.pcbi.1005923

**Published:** 2018-01-09

**Authors:** Wesley Wong, Edward A. Wenger, Daniel L. Hartl, Dyann F. Wirth

**Affiliations:** 1 Department of Immunology and Infectious Diseases, Harvard T. H. Chan School of Public Health, Boston, Massachusetts, United States of America; 2 Institute for Disease Modeling, Bellevue, Washington, United States of America; 3 Department of Organismic and Evolutionary Biology, Harvard University, Cambridge, Massachusetts, United States of America; 4 Broad Institute, Cambridge, Massachusetts, United States of America; University of Chicago, UNITED STATES

## Abstract

Unlike in most pathogens, multiple-strain (polygenomic) infections of *P*. *falciparum* are frequently composed of genetic siblings. These genetic siblings are the result of sexual reproduction and can coinfect the same host when cotransmitted by the same mosquito. The degree with which coinfecting strains are related varies among infections and populations. Because sexual recombination occurs within the mosquito, the relatedness of cotransmitted strains could depend on transmission dynamics, but little is actually known of the factors that influence the relatedness of cotransmitted strains. Part of the uncertainty stems from an incomplete understanding of how within-host and within-vector dynamics affect cotransmission. Cotransmission is difficult to examine experimentally but can be explored using a computational model. We developed a malaria transmission model that simulates sexual reproduction in order to understand what determines the relatedness of cotransmitted strains. This study highlights how the relatedness of cotransmitted strains depends on both within-host and within-vector dynamics including the complexity of infection. We also used our transmission model to analyze the genetic relatedness of polygenomic infections following a series of multiple transmission events and examined the effects of superinfection. Understanding the factors that influence the relatedness of cotransmitted strains could lead to a better understanding of the population-genetic correlates of transmission and therefore be important for public health.

## Author summary

Genomic studies of *P*. *falciparum* reveal that multi-strain infections can include genetically related strains. *P*. *falciparum* must reproduce sexually in the mosquito vector. One consequence of sexual reproduction is that parasites cotransmitted by the same mosquito are related to one another. The degree of genetic relatedness of these parasites can be as great as that of full-siblings. However, our understanding of the cotransmission process is incomplete, and little is known of the role of cotransmission in influencing population genomic processes. To help bridge this gap, we developed a simulation model to determine which of the steps involved in transmission have the greatest impact on the relatedness of parasites cotransmitted by a mosquito vector. The primary goal of this study is to characterize the outcomes of cotransmission following single or multiple transmission events. Our model yields new insights into the cotransmission process, which we believe will be useful for understanding the results from more complicated population models and epidemiological conditions. Such an understanding is important for the use of population genomics to inform public health decisions as well as for understanding of parasite evolution.

## Introduction

Unlike most bacterial and viral pathogens, the malaria parasite *P*. *falciparum*, while predominantly haploid, must sexually reproduce in a mosquito vector before infecting a new human host. Sexual recombination has a significant impact on the population genomics of the parasite, and its effects depend on epidemiological conditions such as transmission intensity [1–3]. One outcome of sexual recombination is that parasites transmitted by a mosquito vector can be genetically related, which can be measured as the proportion of the genome that is identical-by-descent (IBD). IBD segments are region of the genome that originate from a recent common parental strain. A number of studies have used IBD to study transmission [4–8], survey antimalarial resistance [9], and detect signals of selection [10].

The effects of sexual recombination are also apparent in polygenomic (multi-strain) infections. Polygenomic infections can be formed through a series of infectious mosquito bites (superinfection) or through the transmission of multiple strains from a single mosquito bite (cotransmission) [5,7,11]. Coinfecting strains resulting from superinfection are assumed to be unrelated while those resulting from cotransmission are assumed to be genetically related [5–7]. While superinfection is believed to be common in high transmission settings, owing to high entomological inoculation rates and complexity of infections (COI, the number of strains per infection) [12,13], the frequency with which cotransmission occurs is less clear. Studies of genetic relatedness in symptomatic polygenomic infections reporting to clinics in mid-to-low transmission settings show that cotransmission is prevalent in these regions [5–8], but little is known of the frequencies of cotransmission and superinfection across transmission settings. Genetic relatedness studies reveal a large amount of variation in the relatedness of polygenomic infections. The fact that sexual recombination occurs within the mosquito suggests that the relatedness in these polygenomic infections is associated with transmission. High relatedness in polygenomic infections could be indicative of serial cotransmission chains [14], but it is unclear what other factors may influence the relatedness of polygenomic infections.

Part of the uncertainty stems from an incomplete understanding of the cotransmission process. When a female Anopheline mosquito bites an individual infected with malaria, she ingests male and female gametocytes. The ingestion of these gametocytes activates them to form gametes that fuse to create a diploid zygote. Gametes can fuse with other gametes of the same genotype, resulting in self-fertilization (selfing), or can fuse with gametes from other genotypes resulting in outcrossing. The zygote undergoes meiosis and develops into a motile ookinete that traverses the midgut epithelial layer and forms an oocyst. Within the oocyst, the parasite undergoes many rounds of mitosis to create thousands of haploid sporozoites. These sporozoites travel to the mosquito salivary glands and are stored until deposited by the mosquito into the human host during a blood meal. Only those sporozoites that invade the liver will survive to continue the malaria life cycle. How then could variation in within-host and within-vector transmission dynamics, such as the number of oocysts formed and the number of sporozoites infecting the liver, affect the relatedness of cotransmitted strains, and how could these variables in turn affect the relatedness of polygenomic infections in natural populations?

To address the complexity of this transmission cascade and better understand the process of cotransmission, we devised a classification framework based on parasite pedigrees and kinships to develop an understanding of how the various sampling and mating events within the mosquito vector affects the relatedness of transmitted sporozoites. We then created a transmission model to quantify the relatedness of cotransmitted strains under a variety of within-host and within-vector dynamics and used this model to examine the relatedness of polygenomic infections in transmission chains. Our study reveals new insights into the cotransmission process, which we believe will be useful for the interpretation of population genomic signals obtained from more complicated population-level models or from natural populations.

## Results

### Simulating sexual recombination

To simulate sexual recombination, we developed a *P*. *falciparum-*specific meiosis model based on the whole genome sequences of 69 genetically distinct progeny derived from 3 previously generated *P*. *falciparum* crosses involving different laboratory-adapted strains (3D7, HB3, Dd2, 7G8, and GB4) [15–19]. The whole genome sequences generated from these crosses are one of best sources of data for designing a *P*. *falciparum-*specific meiosis model because the genotypes of the parental strains are known. Furthermore, we can be confident of the number of sexual reproduction cycles separating progeny and parental strains. While previous IBD analyses of parasites from natural parasite populations have identified putative F_1_ progeny [5,7,20,21], having complete knowledge of parental ancestry simplifies the identification of IBD segments and allows us to better identify recombination events throughout the genome. We calculated the number of crossover events and inter-crossover distances (S1 Fig & S1 Table) using a hidden Markov model (HMM) [4,22] to identify IBD segments shared between progeny and parental strains (Methods). We then used this data to test the fit of two different meiosis models, one with and one without obligate chiasma formation. Both were based off the gamma model of crossover formation, which has been used to characterize recombination events in a wide variety of taxa, including *H*. *sapiens*, *D*. *melanogaster*, and *S*. *cerevisiae* [23–26]. The gamma model is an improvement over simpler Poisson-based crossover models because it allows us to explore a wide range of crossover interferences.

**Fig 1:**
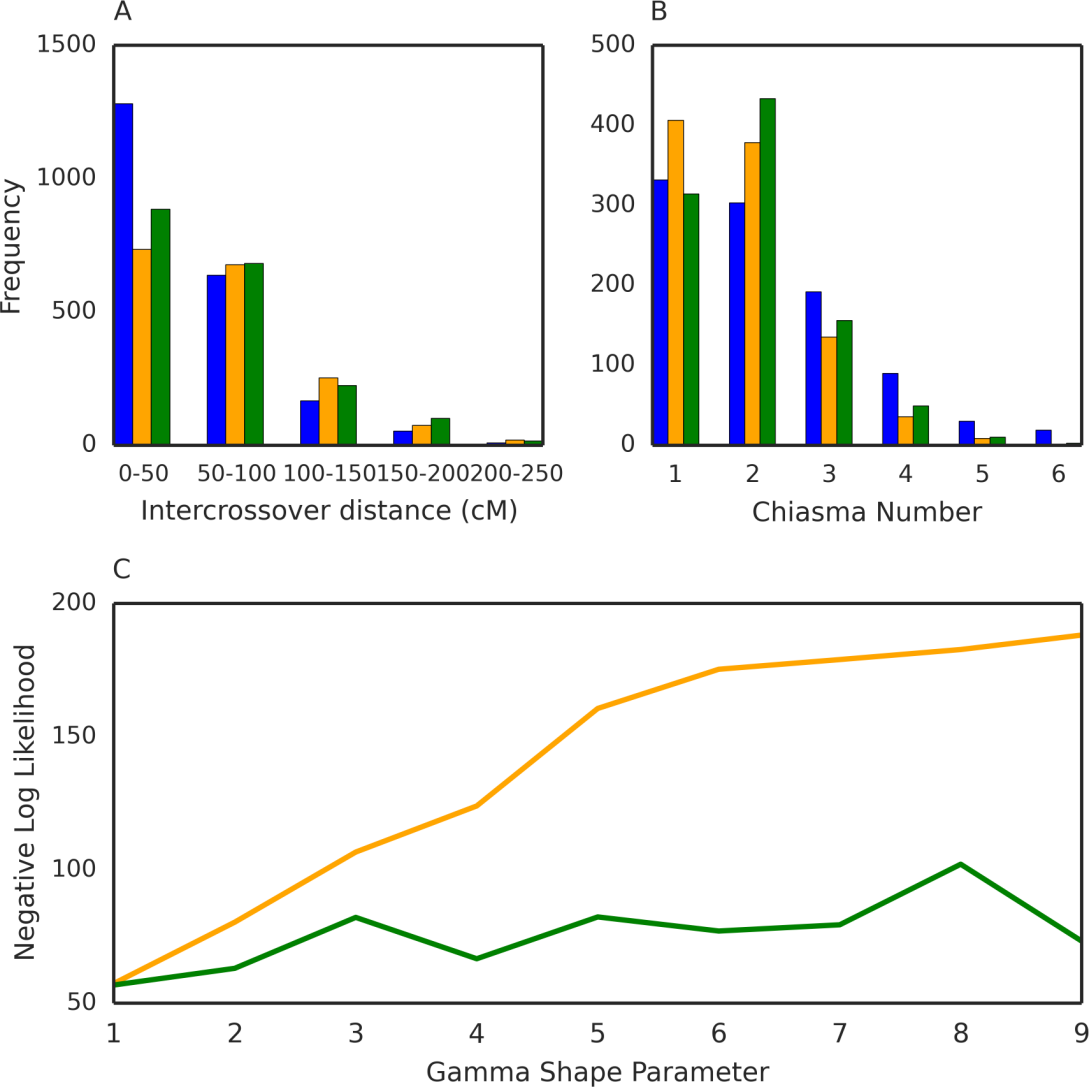
Meiosis simulations. Blue indicates data obtained from the progeny of strains crossed in the laboratory. Orange indicates simulated data using the non-obligate chiasma meiosis model while green indicates simulated data from the obligate chiasma meiosis model. **A**) Barplots of the intercrossover distances of all 14 chromosomes of the *P*. *falciparum* genome. **B**) Barplots of the number of chiasma scattered throughout the genome. For **A** and **B**, simulated data were generated using a shape parameter of 2. **C**) Line plots of the negative pseudo-likelihood values of the non-obligate and obligate meiosis models at different levels of crossover interference. Along the x-axis are different levels of crossover interference (determined by the value of the shape parameter). A shape parameter of 1 indicates no crossover intereference. Lower negative pseudo-likelihood values indicate a better fit to the data obtained from the progeny of experimentally lab-crossed strains.

Regardless of whether obligate chiasma formation was modeled, the number of crossover events and intercrossover distances in our simulated meiotic events resembled those of the laboratory-crossed progeny (Fig 1A and 1B). However, both meiosis models underestimated the frequency of short intercrossover distances (< 50 cM) (Fig 1A), which we suspect is because our HMM overestimated the frequency of short intercrossover distances in the laboratory-cross data (S2 Fig). We found that the obligate chiasma model generated crossover events that were more consistent with that of the laboratory-crossed progeny, but overestimated the number of chromosomes with two crossover events. Using a pseudo-likelihood function (Methods), we determined that an obligate chiasma model fit the data better than a non-obligate chiasma model (Fig 1C). However, we could not estimate the level of crossover interference. Because crossover interference is observed in a wide-variety of organisms spanning multiple taxa [23], we chose to use an obligate chiasma meiosis model with a weak level of interference (gamma distribution with shape = 2, scale = 0.38) for all of our transmission simulations.

#### Role of pedigree and kinship in determining genetic relatedness

To develop an intuition of how the relatedness of cotransmitted strains is influenced by within-host and within-vector transmission dynamics, we developed a framework for understanding how these aspects could affect the relatedness of cotransmitted strains. We reasoned that changes in oocyst counts and COI could alter the relatedness of cotransmitted strains by influencing how gametocytes are sampled and mate within the mosquito vector. This framework is based on one of nine possible pedigrees describing sporozoite pairs. These pedigrees are defined by 1) whether sporozoites are sampled from multiple oocysts and 2) whether these oocysts are the result of selfing or outcrossing. If sporozoites are sampled from multiple oocysts, we consider a third criterion: the number of parental strains shared between oocyst pairs.

**Fig 2:**
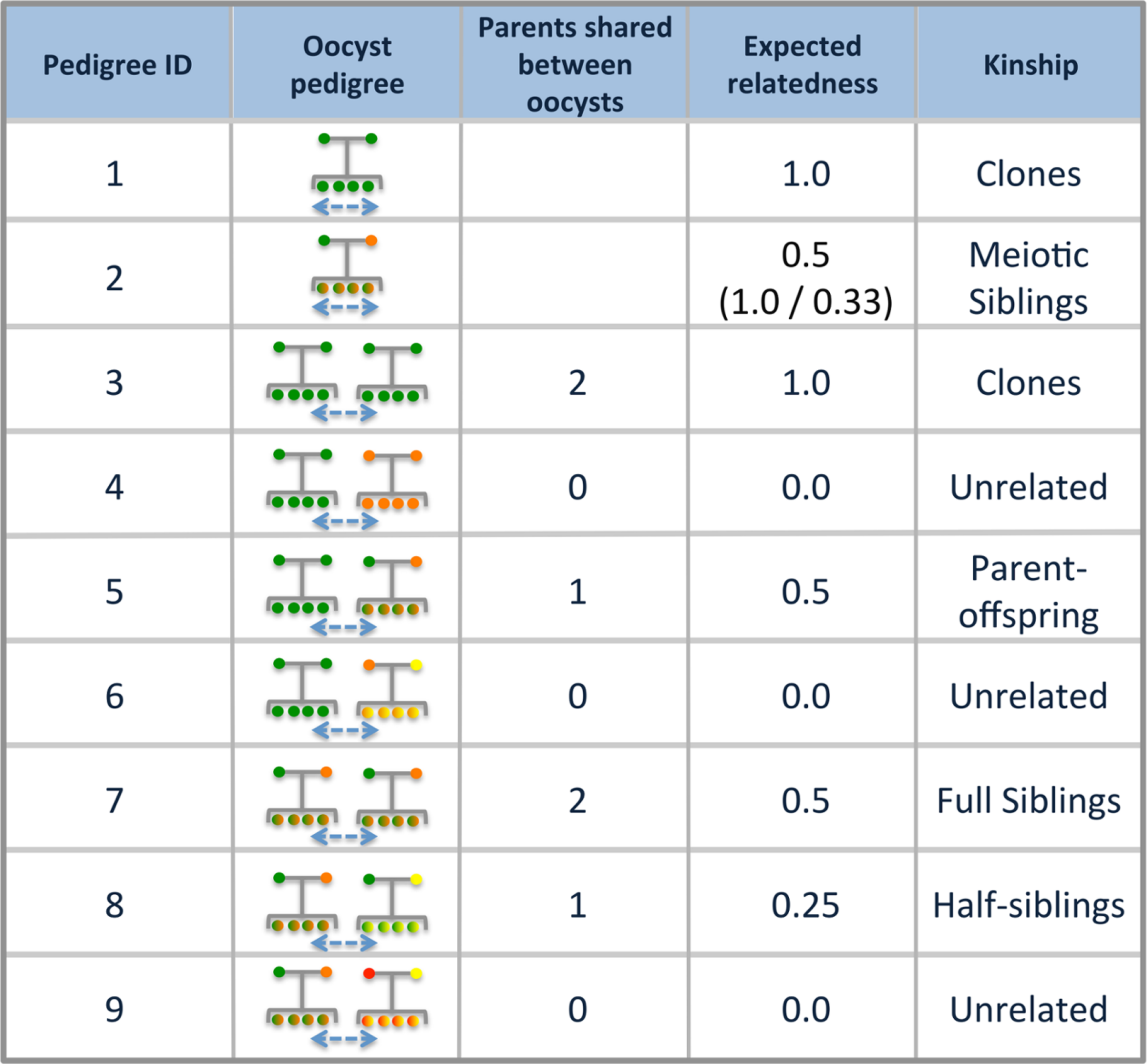
Pedigrees between parasites and their expected relatedness. The 9 possible pedigrees describing parasite pairs. Pedigrees represent the genetic ancestry of parasites and the oocysts they are sampled from. Circles at the top of each pedigree represent the gametes that fuse and undergo meiosis while circles at the bottom represent the sporozoites that are generated following meiosis and expansion in the oocyst. Different colors represent different genomes. Sporozoites with mixed colors indicate they are the result of outcrossing. Blue arrows between pedigrees indicate that sporozoites are sampled from different oocysts. Parasites can be sampled from the same oocyst (pedigrees 1 and 2) or from multiple oocysts (pedigrees 3–9). For pedigree 2, the distribution of expected relatedness was bimodal, and we provide the average across the entire distribution (top) as well as the two modes (bottom). Pedigree 6 and 8 are only accessible when COI ≥ 3 and pedigree 9 is only accessible when COI ≥ 4.

We used our meiosis simulations to quantify the relatedness of sporozoites described by each possible pedigree. Based on the parental ancestries described by our nine pedigrees and the estimate of relatedness provided by our meiosis simulation, we also grouped parasites using kinship definitions. These kinship definitions are analogous to those used in diploid organisms and have been used in other IBD analyses [5]. However, we found that sporozoites sampled from a single, outcrossed oocyst (pedigree 3) could not be described by existing kinship categories. Because they originate from the same meiotic event, we describe their kinship as “meiotic siblings.” Although the average relatedness of meiotic siblings is 0.5, our meiosis simulation revealed that the distribution is bimodal, with one mode at 1.0 (the expected relatedness of genetically identical meiotic siblings) and one mode at 0.33 (the expected relatedness of genetically distinct meiotic siblings) (S3 Fig). Our pedigree/kinship framework and meiosis simulation results are summarized in Fig 2.

### Transmission model description

**Fig 3:**
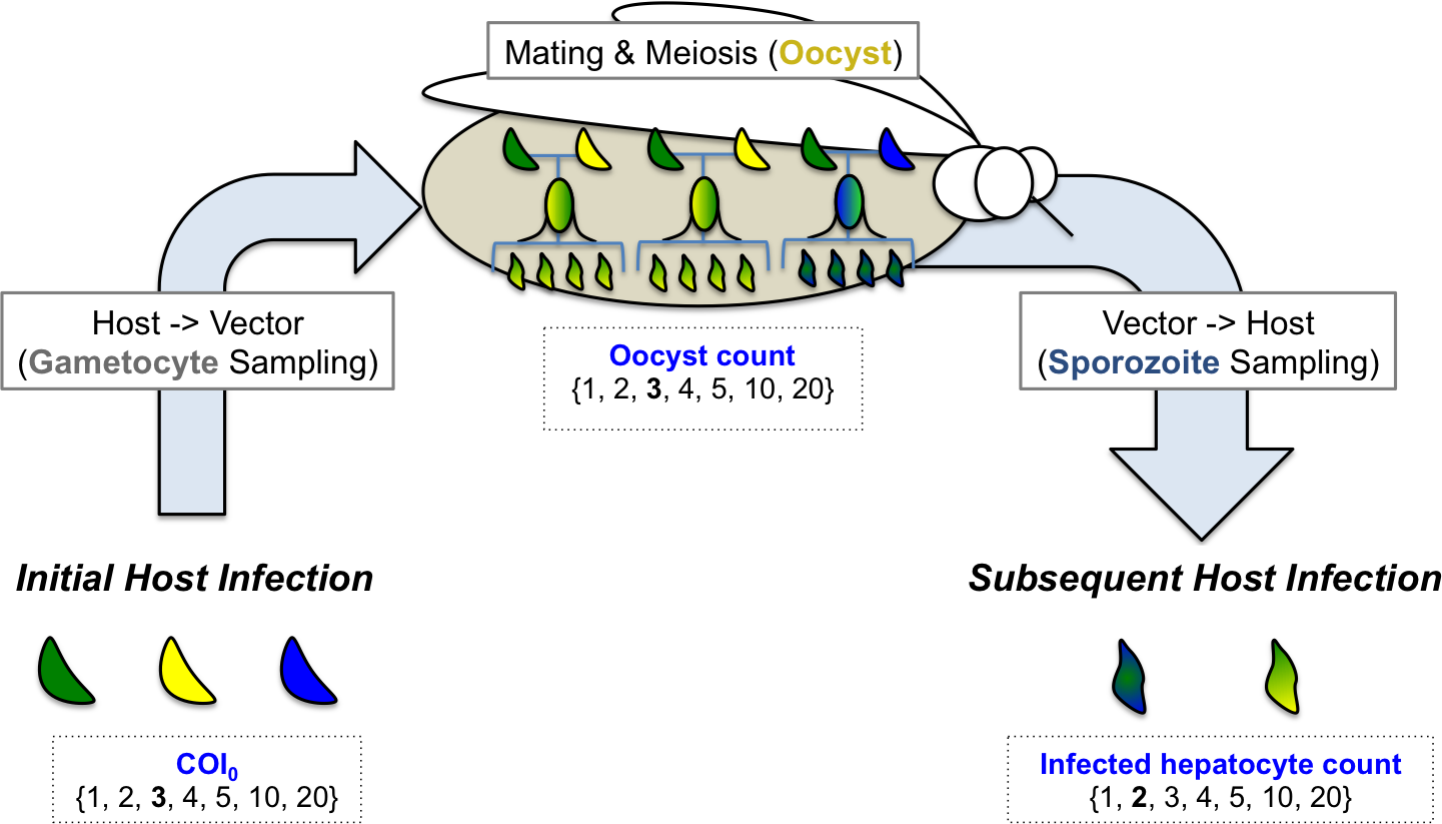
Model of parasite transmission. Model of transmission where genetically distinct parasites are distinguished by color. Parameter values are drawn from the set {1, 2, 3, 4, 5, 10, 20}, which represents the range of values observed in real life simulations. The bold number indicates the value in the example shown in the figure. Gametocytes are sampled from an initial polygenomic infection comprised of unrelated parasite strains. Sampled gametocytes are used to create oocysts within the mosquito midgut (middle). Each oocyst summarizes the entire sequence of events starting from gamete fusion to the end of meiosis. Oocysts are represented using a stylized pedigrees tree, where the crescents at the top represent parental strains undergoing meiosis, the oval in the center indicates whether the mating event is the result of selfing (solid color) or outcrossing (color-gradient), and the sporozoites at the bottom represent the four meiotic products generated through meiosis. Those with multiple colors indicate that genomes have undergone effective recombination and are genetically distinct from the parental strains and to each other. Sporozoites are sampled from the total pool of meiotic products to determine the genetic composition of the subsequent host infection. The number of sporozoites sampled is determined by the infected hepatocyte count used in the simulation.

We then designed a transmission model that partitions transmission into three steps: 1) The host-vector sampling of gametocytes from an initial host infection 2) the sequence of events starting from gamete fusion and meiosis to the development of the oocyst within the mosquito vector, and 3) the vector-host injection of sporozoites and subsequent invasion of the liver to determine the genetic composition of the next human host (Fig 3). We initiate our model by simulating a mosquito blood-feeding event on a polygenomic infection comprised of unrelated strains and parameterized by 1) COI, 2) oocyst count, and 3) the infected hepatocyte count. The number of unique strains present in the initial infection is determined by COI. In our model, we consider oocyst formation as the final outcome of gamete fusion and subsequent meiosis. Based on the oocyst count, our model samples gamete pairs, which fuse and undergo meiosis to create an oocyst consisting of four unique meiotic products. Competition within the oocyst is not modeled and we assume that each meiotic product is present at equal proportion in the oocyst. After all oocysts are created, the model samples sporozoites according to the infected hepatocyte count to determine the genetic composition of the subsequent host infection. If the resulting infection harbors multiple strains, we calculated the relatedness of cotransmitted strains as the average pairwise relatedness between each of the unique genotypes present in the final host infection.

The values for the infected hepatocyte count are pre-specified and drawn from the set {1, 2, 3, 4, 5, 10, 20}. Simulations with COI = 1 were excluded because they always result in selfing and the transmission of genetic clones. Simulations with an infected hepatocyte count = 1 were also excluded, as they cannot result in cotransmission. Small values are overrepresented to reflect the right-skewed distributions of oocyst counts observed in mosquito feeding assays and infected hepatocyte counts estimated from a malaria-challenge study [27–29]. These values also include the COI observed in naturally occurring polygenomic infections from mid-to-low endemic settings (COI ranging from 2–6 in polygenomic infections).

### Single oocyst simulations guarantee the transmission of meiotic siblings or genetic clones

**Fig 4:**
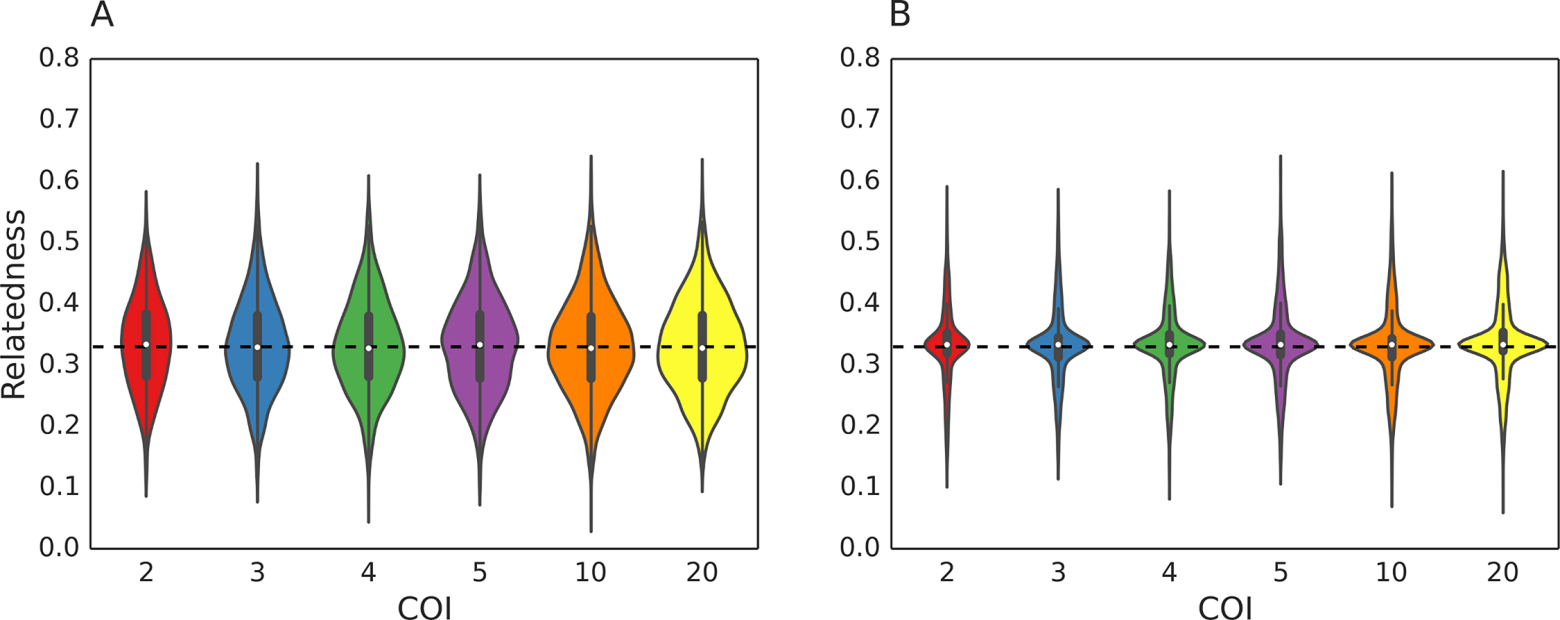
Relatedness of cotransmitted strains when oocyst count is 1. Violin plots of the relatedness of cotransmitted strains in single oocyst simulations. Only the results for simulations with infected hepatocyte count of 2 (**A**) or 5 (**B**) are shown. The expected relatedness (in terms of both median and mean) is always 0.33. A box plot is drawn in the center of each violin plot, where the white dot represents the median of the distribution, the thicker line represent the interquartile range, and the thinner line represents the whiskers of the box plot, up to 1.5 times the interquartile range. The horizontal dotted line represents the value of 0.33.

From our pedigree/kinship framework, we knew that sporozoites sampled from a single oocyst would be either genetic clones or meiotic siblings. Our transmission simulation confirmed this prediction and found that the expected relatedness of cotransmitted strains in single-oocyst transmission simulations was always 0.33 (Fig 4), which is the expected relatedness of genetically distinct meiotic siblings. In single oocyst transmission simulations, cotransmission can only be achieved by the transmission of two or more genetically distinct meiotic siblings. The distinction between genetically distinct and genetically identical meiotic siblings is relevant in the context of cotransmission, as the transmission of clonal meiotic siblings cannot result in cotransmission. Changes to the infected hepatocyte count do not affect the expected relatedness values, but higher infected hepatocyte counts caused the distribution to be more concentrated around the mean.

### The expected relatedness of cotransmitted strains in multiple oocyst simulations depends on COI

In multiple oocyst transmission simulations, the relatedness of cotransmitted strains is not as easy to predict, since multiple kinships can be transmitted. Based on our pedigree/kinship framework, we hypothesized that COI modulates the expected relatedness of cotransmitted strains by limiting the transmission of half-siblings and unrelated strains; the transmission of half-siblings and unrelated strains described by pedigrees 6 are only possible when COI ≥ 3. The transmission of unrelated strains described by pedigree 9 only applies when COI ≥ 4.

**Fig 5:**
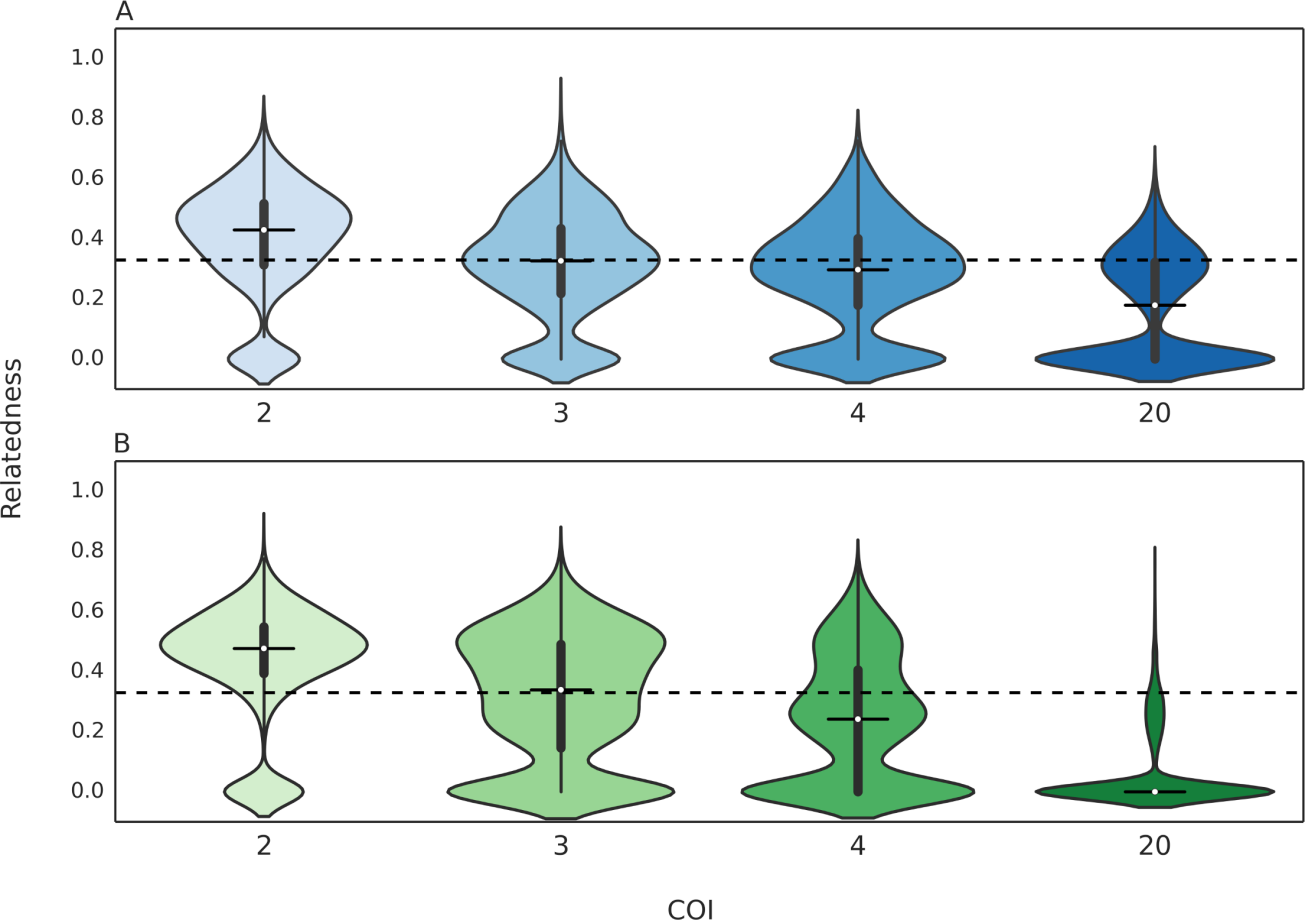
Relatedness of cotransmitted strains in multiple oocyst simulations. Violin plots of the relatedness of cotransmitted strains in multiple oocyst simulations. Only the results for simulations with oocyst counts of 2 (A) and 20 (B) and infected hepatocyte counts of 2 are shown. The expected relatedness of cotransmitted strains declines with increasing COI. A box plot is drawn in the center of each violin plot, where the white dot represents the median of the distribution, the thicker line represent the interquartile range, and the thinner line represents the whiskers of the box plot, up to 1.5 times the interquartile range. The horizontal dotted line represents the value of 0.33.

**Fig 6:**
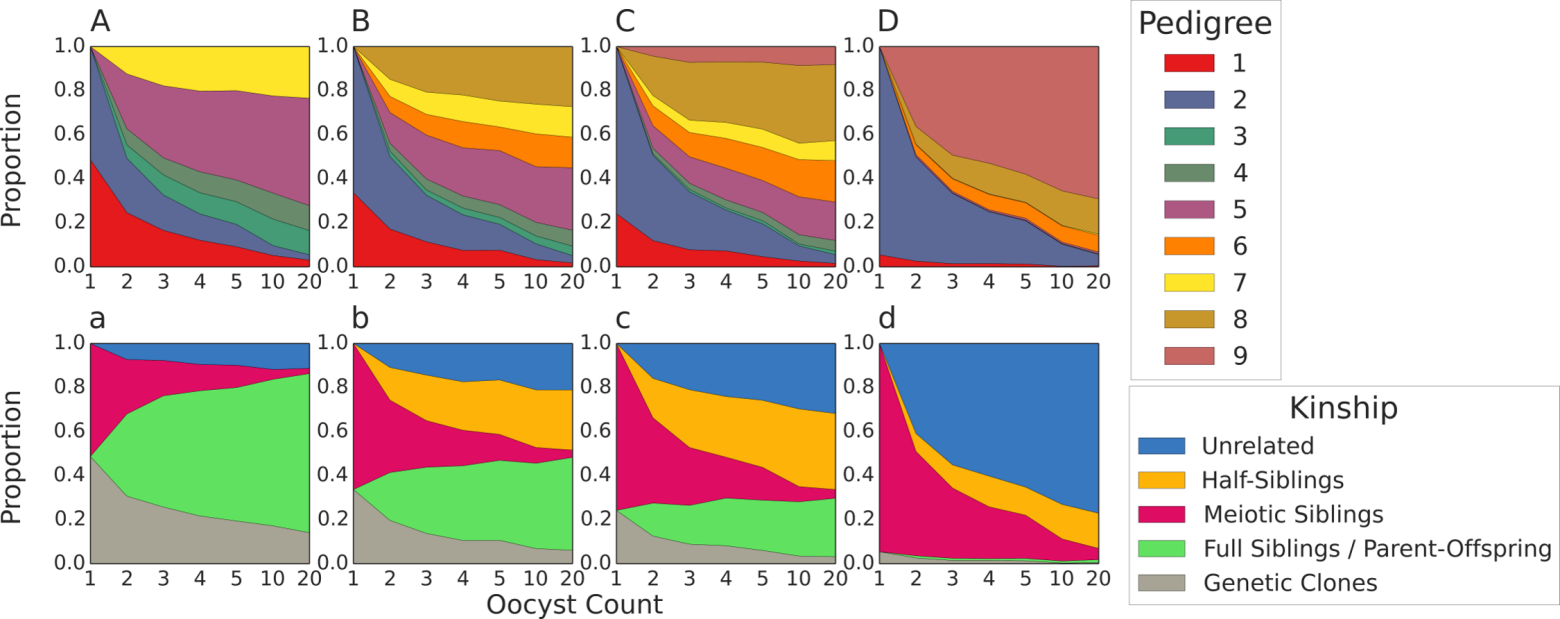
Pedigree and kinship frequencies from multiple oocyst simulations. Stacked line charts of the frequencies of different pedigrees (A-D) and kinships (a-d) plotted against oocyst count. Each subplot represents a scenario with a different COI (A/a = 2, B/b = 3, C/c = 4, D/d = 20). Only the results from simulations where infected hepatocyte count = 10 are shown. Genetic clones are defined as those emerging from oocysts characterized by pedigree 1 and 3; genetically identical meiotic siblings are still classified as meiotic siblings in this graph.

Our transmission simulations confirmed these predictions and revealed a simple relationship between COI, oocyst count, and the relatedness of cotransmitted strains (Figs 5 and 6): the relatedness of cotransmitted strains declines with increasing COI. All COI = 2 simulations have an expected relatedness > 0.33, with a larger increase in high oocyst count simulations. The increase in relatedness is a reflection of the increased transmission of full-siblings and parent-offspring strains. When COI = 3, increasing oocyst counts no longer increased the expected relatedness of cotransmitted strains due to the additional transmission of half-siblings. Once COI > 4, increasing oocyst counts decreased the expected relatedness of cotransmitted strains. This was due to the increased transmission of unrelated strains, particularly those described by pedigree 9 (outcrossed oocysts that do not share any parental strains) (Fig 6C and 6D). When COI = 20, the majority of transmitted parasites are either meiotic siblings or unrelated strains described by pedigree 9.

We found that different infected hepatocyte counts altered the distribution of relatedness (S4 Fig & S5 Fig) but had no effect on the trends established by either COI or oocyst count. Again, simulations with a COI = 2 consistently had the highest expected relatedness values while simulations with higher COIs had lower expected relatedness values, regardless of the infected hepatocyte count.

### Investigating the effect of non-uniform gametocyte sampling probabilities

Thus far, our simulations have assumed that the strains making up polygenomic infection are present and sampled in equal proportions. However, strain proportions in natural polygenomic infections can be highly skewed. Furthermore, different strains can have different transmissibility relating to factors such as gametocyte production. To investigate how skewed gametocyte sampling probabilities could affect the relatedness of cotransmitted strains, we devised a weighted sampling scheme defined by the ratio of the most frequent to the least frequent strain in the infection (**Methods**).

Predictably, skewing the gametocyte strain ratios increased the rate of selfing and the transmission of genetic clones (S6 Fig). Skewed ratios of up to 10:1 increased relatedness of cotransmitted strains by a small amount. Ratios ranging from 1:1 to 10:1 increased the expected relatedness of cotransmitted strains by 0.01–0.10. This increase depended on both COI and the magnitude by which strains proportions differed. The relatedness of cotransmitted strains from high COI infections was more robust to differences in strain proportions; a 10:1 ratio in a COI = 20 infection increased relatedness by only 0.02 while a 10:1 ratio in a COI = 3 infection increased relatedness by 0.03–0.06.

### Generating a combined model of cotransmission and examining serial cotransmission chains

The genetic composition of natural polygenomic infections can result from multiple transmission events and influenced by population-level transmission dynamics. However, developing a model that take into account all possible population-level transmission dynamics is beyond the scope of this paper. Instead, we used our model to quantify the relatedness of polygenomic infections in three different multiple transmission simulations, which we refer to as transmission lineages. Each transmission lineage is designed to resemble transmission chains that occur in natural populations and initiated by simulating a mosquito blood-feeding event on a polygenomic infection comprised of unrelated strains. The first transmission lineage does not allow superinfection; all subsequent transmission events in the chain must infect uninfected hosts. The second and third transmission lineages allow superinfection and are differentiated by the nature of the resident strain in the soon-to-be superinfected host. For the second transmission lineage, the resident strain is identical to one of the parental strains in the initial polygenomic infection (resembling natural backcrossing events). For the third transmission lineage, the resident strain is not related to any of the parental strains in the initial polygenomic infection but is the same in all transmission events. In the last transmission lineage, the resident strain is not related to any of the parental strains in the initial polygenomic infection and is different in all transmission events.

For our transmission lineage simulations, we modified our cotransmission model so that oocyst and infected hepatocyte counts are determined by randomly sampling from distributions reflecting those of found in previous studies [29,30]. Subsequent transmission events sample parasites from the infection generated by the previous transmission event. Allowing oocyst and infected hepatocyte counts to be chosen from these distributions did not affect the previously observed relationship between COI and the relatedness of cotransmitted strains (S7 Fig). The relatedness of cotransmitted strains following single cotransmission events from infections COI = 2 had an expected relatedness greater than 0.33 while those with a COI > 3 had an expected relatedness less than 0.33.

**Fig 7:**
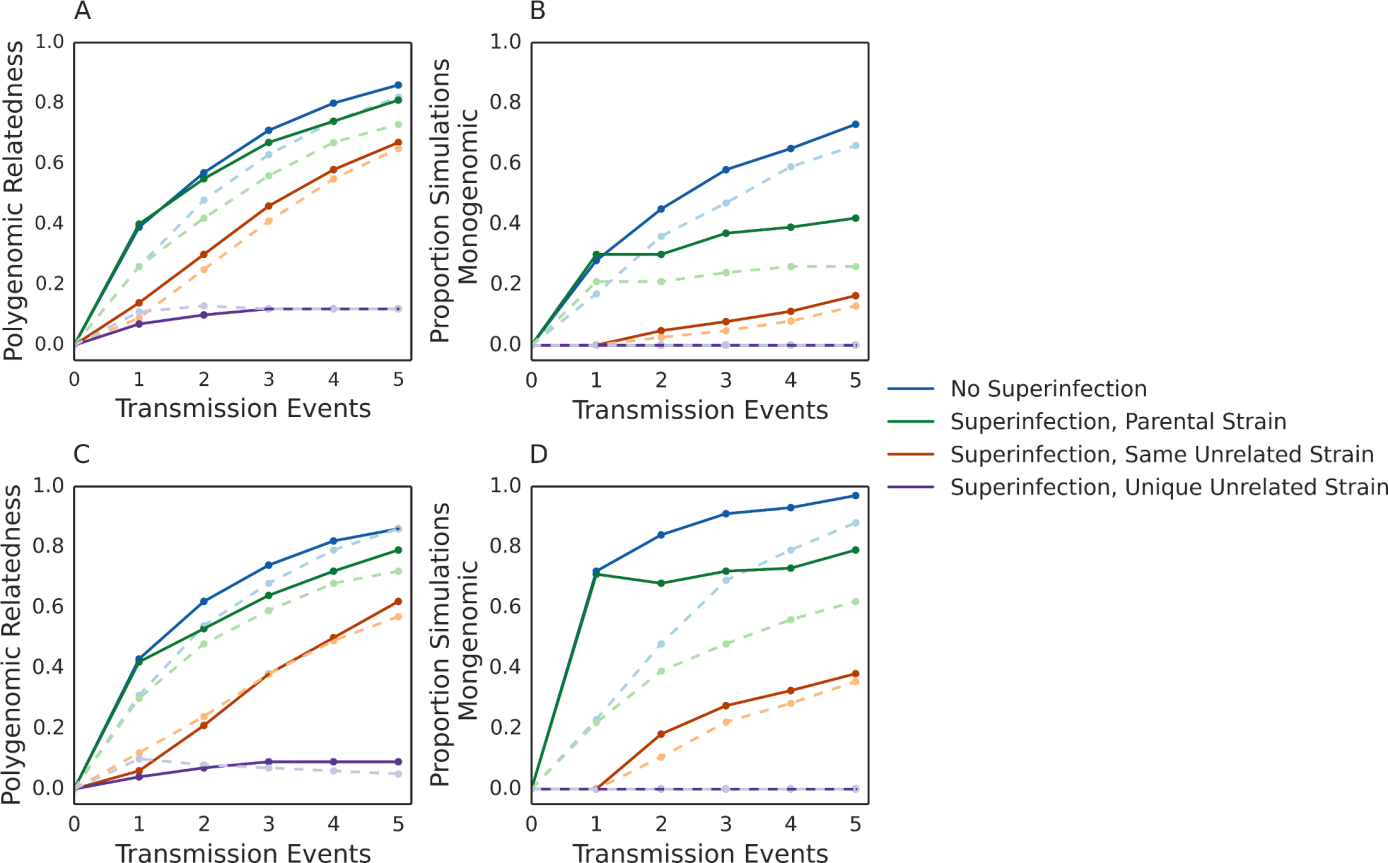
Relatedness of polygenomic infections after multiple transmission events. Line plots of the average relatedness of polygenomic infections **(A,C)** and proportion of simulations that have converged to the transmission of a single strain after multiple transmission events across 500 simulations **(B,D)**. Only results from simulations where gametocyte-sampling probabilities are equal **(A,B)** and where gametocyte sampling probabilities are skewed by a 10:1 ratio between the most frequent and least frequent strain **(C,D**) are shown. Blue = No superinfection. Green = Superinfection where the resident strain is the same as one of the parental strains in the initial infection. Red = Superinfection where the resident strain is unrelated to the parental strains in the initial infection. Purple = Superinfection where the resident strain is unrelated and different in each transmission event. Solid dark lines indicate results where the initial polygenomic infection had a COI = 2 and light dotted lines indicate results where the initial polygenomic infection had a COI = 5.

As expected of serial cotransmission chains, we found that the relatedness of polygenomic infections increases with each transmission event (Fig 7). Transmission lineages with superinfection had lower relatedness values and smaller proportions of serial transmission simulations that converged to the transmission of single strains. The reduction in relatedness was greatest in those where the resident strain was unrelated to the parental strains of the original infection (Fig 7, purple). Changing the resident strain after each transmission event prevented the relatedness of polygenomic relatedness from increasing beyond 0.10 even after five transmission events. We also saw that the COI of the initial infection could have a lasting effect on the relatedness of polygenomic infections. Transmission lineages initiated with low COI polygenomic infections had higher relatedness values than those initiated with high COI polygenomic infections. This effect was weaker in superinfection lineages with unrelated resident strains. While skewed gametocyte-sampling ratios had a modest effect on the relatedness of polygenomic infection, it drastically increased the rate with which transmission lineages converged to the transmission of single strains for all transmission lineages except the one where unrelated resident strains were changed after each transmission event (Fig 7B and 7D).

## Discussion

Parasite strains in polygenomic infections are often genetically related, but it is unclear why there is so much variation between infections or whether the relatedness of polygenomic infections can be used to understand parasite transmission. In order to help bridge the gaps in our understanding, we developed a pedigree/kinship framework for understanding how COI and oocyst counts affect the relatedness of cotransmitted strains. We then tested the predictions of this framework using a parasite transmission model to quantify changes in the relatedness of cotransmitted strains. We demonstrated that multiple oocyst simulations in low COI conditions favor the transmission of full-siblings / parent-offspring strains and limit the transmission of half-siblings and unrelated strains, causing an increase in the expected relatedness of cotransmitted strains. Multiple oocyst simulations in high COI conditions decrease the relatedness of cotransmitted strains by favoring the transmission of half-siblings and unrelated strains. Alterations to the number of sporozoites that invade the liver have little effect on relatedness, conditioned on the fact that multiple sporozoites invade.

We also examined how non-uniform gametocyte-sampling probabilities could affect the relatedness of cotransmitted strains. Previous studies have established that intra-host parasite dynamics depend on patient age [31,32] disease severity (reviewed in [33]), and eco-epidemiological factors such as seasonal transmission [34,35]. These dynamics are strongly influenced by host immunity [36] and can fluctuate over the course of a single infection [32,37–40]. Furthermore, gametocyte sampling is not completely random [41] and not reliant on peripheral blood gametocyte densities at low parasitemias [34,42]. Our results show that the relatedness of cotransmitted strains is robust to variations in intra-host strain proportions and gametocyte-sampling probabilities. Even infections where the ratio of the most frequent to least frequent strain is 10:1 do not result in drastic changes to that observed from infections with even strain proportions. This suggests that the relatedness of cotransmitted strains is consistent across differences in patient-age, disease severity, and host immunity.

Our results are in agreement with the frequent assumption that cotransmission events are comprised of genetically related parasite strains [5–8]. A large fraction of simulated cotransmission events result in the transmission of genetically distinct meiotic siblings, as evidenced by the peaks at 0.33 for all simulations where oocyst counts and hepatocyte counts were randomly sampled. However, we also found that the transmission of unrelated strains is a major aspect of cotransmission. The cotransmission of unrelated strains was present in all multiple oocyst simulations and increased in frequency with COI. Polygenomic infections comprised of unrelated strains are typically assumed to be the result of superinfection, but these findings suggest that some are the result of cotransmission. Current estimates of the prevalence of cotransmission are underestimates, since they rely on the subset of cotransmission events resulting in polygenomic infections comprised of genetically related strains [7].

Our results reveal an inverse relationship between the relatedness of cotransmitted strains and COI. COI is correlated with high entomological inoculation rates [43,44] and a known genetic correlate of transmission intensity [43,44]. COI is higher in high transmission areas than in low transmission areas due to increased superinfection rates. The association between the relatedness of cotransmitted strains and COI suggests that polygenomic infections in low transmission areas are comprised of more related strains than those in high transmission areas. We previously found that the average relatedness of 32 symptomatic polygenomic patients collected from a clinic in a low transmission region of Senegal (mean COI of two) was 0.38 [7]. This value exceeds the expected relatedness of meiotic siblings and may reflect an increase in the transmission of full-siblings / parent-offspring parasites but could also result from factors such as population structure. Previous studies of genetic relatedness have focused on areas of mid-to-low transmission setting [5–8] and a comparison of genetic relatedness of polygenomic infections across transmission settings have yet to be performed. High relatedness from low COI infections could have implications for the spread of drug resistance traits in low transmission settings, as the increased relatedness could increase the chance that multi-locus drug resistant genes are passed on together to the next generation.

It remains to be seen whether the relationship between relatedness and COI can be reflected in polygenomic infections collected from natural parasite populations. If the inverse relationship between COI and relatedness holds, then the relatedness of coinfecting strains could be a potential population genetic correlate of transmission intensity. Population genetic correlates of transmission are valuable in the context of malaria control and can be used to supplement or supplant traditional epidemiological measures, which can be difficult to collect in low transmission areas [44,45]. With regards to polygenomic infections, only the frequency and COI of polygenomic infections are known to correlate with transmission intensity [4,44,46]. Other population genetic metrics, such as parasite clonality [44], currently rely on data obtained from monogenomic infections, which are limited in high transmission areas where polygenomic infections are frequent. By providing an additional source of information, genetic relatedness could increase the granularity by which we use genetic signals to monitor changes in transmission. However, spatial-temporal transmission, such as the seasonality or the existence of transmission hotspots, and host immunity can influence population genetic structure [36]. Neither of these are taken into consideration in this study, and it is unclear how these might affect polygenomic relatedness. Population-level models and epidemiological sampling will be needed to understand the effects of cotransmission and establish whether the relatedness of polygenomic infections correlates with transmission intensity.

An alternative method of dissecting population-level dynamics is to focus on the characterization of transmission lineage. Transmission lineages consist of chained transmission events and are a simplification of the transmission processes within populations. Our transmission lineages were designed to examine the effect of multiple transmission events and to examine how the co-occurrence of superinfection affects the relatedness of polygenomic infections. They show that superinfection depresses the relatedness of polygenomic infections, but also show how sensitive these lineages are to the conditions of the host infection. Strikingly, they show that cotransmission fails to increase the relatedness of polygenomic infections if each host in the transmission chain harbors a different, genetically unrelated parasite strain. They also reveal the fragility of serial cotransmission chains. In the absence of superinfection, serial cotransmission chains quickly converge to the transmission of single strains. High COI in the initial infection delays this process but a large fraction of serial cotransmission chains still converge within five transmission events. Because these transmission lineages are analogous to the introduction of a polygenomic infection to a new population, polygenomic relatedness could be useful for studying transmission in import scenarios.

In conclusion, our study uses a model of parasite transmission to provide mechanistic insight into the process of cotransmission to help understand the factors that influence the relatedness of cotransmitted strains. Understanding the effects of sexual recombination and transmission on malaria population genomics is of key public health interest in an era where parasite populations are experiencing rapid declines in transmission intensity. We believe mechanistic models such as the one used in this study reveal new insights that can be applied to the results obtained from more complicated conditions. Our model highlights the importance of COI in influencing the relatedness of cotransmitted strains, but future models and epidemiology studies are needed uncover how transmission intensity and cotransmission affects the genetic composition of strains in polygenomic infections in natural populations. These models should incorporate background parasite population structure and genetic diversity to understand the effects of cotransmission and establish whether the relatedness of polygenomic infections correlates with transmission intensity. Such models will rely on genetic data collected from well-characterized epidemiological settings to determine whether the relatedness of polygenomic infections is a potential population genetic correlate of transmission.

## Methods and models

### Simulating meiosis and recombination

We simulated meiosis under two different frameworks: one with and one without obligate chiasma formation. Both frameworks sample from a constrained gamma distribution where the average distance between randomly sampled distances is 50 centimorgans to determine the location of chiasma along a bivalent [47,24]. For each placed chiasma, our meiosis model chose one sister chromatid from each homolog to undergo recombination. Sister chromatids were independently chosen for each recombination event. Once all recombination events were complete, the model independently segregated and randomly combined sister chromatids from other bivalents to create haploid parasite genomes.

For the non-obligate chiasma framework, our meiosis model placed the first chiasma 10^5^ base pairs before the beginning of each chromosome. It then drew a distance, *d*, from a gamma distribution with shape *= v* and scale = 1/(2*v*) [47] to determine the location of the next chiasma. New chiasma were placed *d* units after the previous chiasma and a new distance was drawn for each chiasma. Chiasma locations were filtered to include only those that fell within the boundaries of the chromosome under consideration.

For the obligate chiasma framework, the position of the first chiasma was determined by drawing from a uniform distribution that spans the length of each chromosome. Subsequent chiasma were placed by drawing distances from a constrained gamma distribution (described in the next paragraph) and placing the next chiasma *d* units before it. This was repeated until the start of the chromosome was reached. Afterwards, the process was repeated in the other direction until the end of the chromosome was reached.

Due to the forced placement of chiasma, we could not use the formulas used in the non-obligate chiasma framework to generate appropriately constrained gamma distributions. We used an approximate Bayesian computation (ABC) Markov chain Monte Carlo (MCMC) to solve the appropriate scale parameter and shape parameters. Shape parameters varied from 1–9 and scale parameters were sampled from a uniform distribution with a range of 0–5. For each set of scale and shape parameters, we counted the number of chiasma on a bivalant 100 centiMorgans (cM) in length and repeated this process 1000 times to estimate the average and standard deviation. We evaluated the fit of each proposed parameter using the following distance metric:

$$D\prime =\frac{{\left(2-u\right)}^{2}}{0.05^{2}+\delta^{2}}$$

where *u* and δ are the simulated mean and standard deviations of the number of chiasma, 2 represents the desired number of chiasma per 100 centiMorgans, and 0.05 represents a small error term. We then constructed an estimate of the pseudo-likelihood as:

$$L=\frac{1}{e^{D^{\prime }}}$$

The proposed scale parameter was accepted if the proposed pseudo-likelihood was greater than the pseudo-likelihood of the previously proposed parameter. If the new pseudo-likelihood was smaller, then the probability of rejection was decided by the ratio of the current pseudo-likelihood over the previous pseudo-likelihood. This process was repeated 2,500 times to form a MCMC chain. After our MCMC chain was completed, we calculated the mean of the accepted scale parameters from the last 1500 steps to serve as our estimate of the scale for each shape parameter.

### Model selection: Non-obligate vs obligate chiasma model

We calculated the average number of crossover events and intercrossover distances for each chromosome in the genome using SNP data from 69 genetically distinct progeny generated from 3 different laboratory crosses [15,17–19]. These data were previously generated by the Pf3k project (https://www.malariagen.net/projects/pf3k) [15–19]. VCF files were downloaded and filtered based on the available INFO strings. We removed non-Mendelian sites, sites that did not pass the quality filters used, and sites that were invariant between the parental strains used in the cross. Samples from each laboratory cross were represented by an average of 1028 SNPs. From this filtered dataset, we performed pairwise calculations of percent similarity to identify and remove duplicate strains. Duplicate strains were defined as those having greater than 90% SNP similarity.

For each chromosome, we used a modified version of an IBD Hidden Markov Model (HMM) [4,22] to quantify the average number of crossover events and the average intercrossover distance for each chromosome. Our previously published HMM relied on population SNP frequencies to infer IBD, which is problematic when using cultured strains with vague demographic histories. For each laboratory cross, we used SNP data to infer IBD between progeny and parental strains using the following emission probabilities:

$$P(Concordance|IBD)={\left(1-\varepsilon \right)}^{2}+{\left(\varepsilon \right)}^{2}$$

P(Concordance|non−IBD)=2ε(1−ε)

P(Discordance|IBD)=2ε(1−ε)

P(Discordance|non−IBD)=1−2ε(1−ε)

where ε refers to the rate of sequencing error, concordance refers to having the same SNP identity, discordance refers to having different SNP identities, and IBD refers to identical-by-descent.

The resulting IBD maps closely mirror the parental inheritance boundaries specified in [16], but sometimes identifies very short IBD fragments that are unlikely to be real (S5 Fig). Crossover events were identified as the points in the chromosome where the IBD map switches from IBD to non-IBD and intercrossover distance was calculated as the distance (in cM) between each of the identified crossover points. Intercrossover distances were converted to centiMorgans using the estimates reported in [15,16] (15 kb/cM). If no crossovers were observed, then the intercrossover distance was defined as the length of the entire chromosome.

We then used the average number of crossovers and intercrossover distances to determine whether a non-obligate or obligate chiasma model of meiosis would fit the data better. Each simulation was run 20 times to get an average and standard deviation of the number of crossover events and crossover distances per chromosome. We then devised a distance metric defined as:

$$D_{j}=\sum_{i}^{14}\frac{{\left(u_{i,sim}-u_{i,observed}\right)}^{2}}{{\delta^{2}}_{i,sim}+{\delta^{2}}_{i,observed}}$$

where *u* is the mean, δ is the standard deviation, *j* is the feature (number of crossover events or intercrossover distance), *i* is the chromosome number, *sim* indicates the simulation result, and *observed* indicates the value observed in the 69 progeny strains. We defined a pseudo-likelihood as

$$L=\prod_{j}^{2}\frac{1}{e^{D_{j}}}$$

and used it to determine the model that fit the data better.

### Model design: Modeling transmission

To quantify the average relatedness of cotransmitted strains, we developed an agent-based mosquito transmission model that simulates the sampling processes that occur as parasites enter and exit the mosquito vector and parameterized by COI, oocyst count, and infected hepatocyte count. The values for oocyst count and infected hepatocyte count were drawn from the set {1, 2, 3, 4, 5, 10, 20} while the values for COI were drawn from the set {2, 3, 4, 5, 10, 20}. Each set of parameters was run 2000 times. Each simulation was initiated by creating an initial infection comprised of unrelated parasite strains; the number of strains within the initial infection was determined by COI.

To model differences in intra-host strain proportions and differences in sampling probabilities, we assumed that strain proportions followed an exponential equation of the form:

$$f(x)=Ae^{Bx}$$

where x is a discrete variable representing each strain in the infection. We used an exponential equation to magnify the difference in frequency between the most frequent strain and the other strains present in the infection.

For an infection with COI = *n*, *x* ranges from 0 to *n -1*. We fit this equation to two points, (0, *f*(0)) and (*n—1*, *f*(*n—1*)), based on the ratio of the most frequent to the least frequent strain in the infection. These ratios ranged from 1:1 to 10:1, reflecting the observed strain proportions in a set of polygenomic infections collected from Thiès, Senegal (S8 Fig). *f*(0) is the ratio of the most frequent to the least frequent strain. *f*(*n—1*) is the ratio of the final strain to the least frequent strain and always equal to one. The ratios of all other strains present in the infection was determined by *f*(1), *f*(2), …*f*(*n*-1). We then drew from a Dirichlet distribution with a concentration parameter = {*f*(0), *f*(1), *f*(2), … *f*(*n—*1)} 1000 times to calculate the expected frequency of each strain in the infection.

Based on the specified oocyst count, our model sampled gametocyte pairs by their intra-host strain proportions to create oocysts, allowing for multiple samplings of the same strain. Each sample pair underwent meiosis to create four meiotic products. The progeny from all the meiotic events were combined without the removal of repeat strains to represent the sporozoites within the mosquito vector. Our model assumed mating success and oocyst formation could be simulated as the random sampling of gametocytes from the human host. It is unclear whether the parasite has a preference for self-fertilization or outcrossing. Evidence for non-random mating is based on the observation of highly inbred oocysts within the mosquito midgut [48], but it is unknown to what extent self-fertilization occurs more frequently than expected by chance.

We then sampled sporozoites to represent the strains in the infected hepatocytes. Multiply-infected hepatocytes were not allowed. At this point, our model performed pairwise comparisons between all the parasites in the infected hepatocytes, regardless of whether or not the pair consisted of genetically distinct parasites, to determine the frequency of the different pedigrees specified in Fig 2. The expected relatedness of cotransmitted strains was calculated as the average pairwise relatedness between genetically distinct strains. This average is not weighted by the frequency of strains within the infected hepatocytes. Because cotransmission must result in the creation of polygenomic infections, we excluded infections where the infected hepatocytes consisted of a single strain. When an infected hepatocytes consisted of two or more genetically distinct strains, the relatedness of cotransmitted strains was calculated as the relatedness between the two strains; when an infection was comprised of 20 genetically distinct strains, the relatedness of cotransmitted strains is calculated as the average pairwise relatedness from all 20-choose-2 comparisons.

Source code is available on GitHub, under the project name Cotransmission (https://github.com/weswong/Cotransmission). The code is written using Python 2.7.0 and is platform independent.

### Quantifying relatedness in simulated genomes

We defined relatedness as the proportion of the genome that is identical-by-descent (IBD) owing to inheritance from the same common ancestor. Because the genetic ancestry of all input strains was known and assumed to be genetically unrelated, IBD segments were identified as segments of the genome that originated from the same parental input strain.

### Calculating the expected relatedness of the nine pedigrees

To calculate the expected relatedness of parasites described by our 9 pedigrees, we generated simulations with the appropriate number of oocysts (1 or 2), the appropriate pedigreess for each oocyst, and the appropriate method of sampling parasite pairs (within or between oocysts) for each pedigree and quantified the relatedness of a single randomly drawn parasite pair. This process was repeated 800 times to generate distributions of relatedness and to get an estimate of the mean.

## Supporting information

S1 Fig Observed intercrossover distances from progeny of lab crossed strains. Histograms of the distribution of intercrossover distances (cM) for each chromosome in the *P*. *falciparum* genome. Dark blue indicate distances whose boundaries fall within each chromosome and light blue represents distances that span the entire chromosome.
(TIF)

S2 Fig IBD map comparison. Comparison of the parental inheritance boundaries defined by [16] (left) and our HMM (right) for A) 3D7_ERR019061 (parental) vs C12_ERR019063 (progeny) B) 7G8_ERR027099 (parental) vs AUD_ERR029406 (progeny) and C) DD2_ERR012840 (parental) vs 3BA6_ERR126027 (progeny). For the maps based on the boundaries defined by [16], only the results from chromosomes with evidence of recombination are shown. Orange coloration indicates a section of the genome inherited from 3D7_ERR019061, 7G8_ERR027099, or DD2_ERR012840 while grey sections indicate a section inherited by the other parent in the cross. Our HMM occasionally identified short IBD segments (marked with arrows) not present in the data from [16].
(PNG)

S3 Fig Relatedness distributions for each of the 9 pedigrees. Histograms of the expected relatedness for each pedigree. Orange: Relatedness of between progeny strains. Yellow: relatedness of progeny strains vs one of the parental strains. The blue dotted line represents the expected relatedness of half-siblings (0.25), the green dotted line represents the expected relatedness of unique meiotic siblings (0.33), and the purple dotted line represents the expected relatedness of full-siblings / parent-offspring strains (0.5).
(TIF)

S4 Fig Relatedness of cotransmitted strains in multiple oocyst simulations with high infected hepatocyte counts. Violin plots of the relatedness of cotransmitted strains in simulations where the infected hepatocyte count was 20 and the oocyst count was 2 (**A**) or 20 (**B**). A box plot is drawn in the center of each violin plot, where the white dot represents the median of the distribution, the thicker line represent the interquartile range, and the thinner line represents the whiskers of the box plot, up to 1.5 times the interquartile range. The horizontal dotted line represents the value of 0.33.
(TIF)

S5 Fig Pedigree and kinship frequencies from multiple oocyst simulations with high infected hepatocyte counts. Stacked line charts of the frequencies of different pedigrees (A-D) and kinships (a-d) plotted against oocyst count. Each subplot represents a scenario with a different COI (A/a = 2, B/b = 3, C/c = 4, D/d = 20). Results from simulations where infected hepatocyte count = 20 are shown. Genetic clones are defined as those emerging from oocysts characterized by pedigree 1 and 3; genetically identical meiotic siblings are still classified as meiotic siblings in this graph.
(TIF)

S6 Fig Non-uniform gametocyte sampling probabilities. Strain frequencies in COI 2 (A), 4 (B) and 20 (C) infections. We examined strain proportions ranging from 1:1 to 10:1 for all COI infections. We also examined a 1000:1 ratio for COI = 20 infections. Ratios exceeding 10:1 were not examined in the COI = 2 and COI = 4 infections because the minor strains become so infrequent that the infections could be considered lower COI infections. **B**) Expected relatedness of cotransmitted strains after a single cotransmission event. Only results using oocyst and infected hepatocyte counts of 2 are shown. **C**) Kinships among transmitted parasites from infections with different strain proportions. Only results using oocyst and infected counts of 2 from a COI = 2 infection are shown. **D**) Expected relatedness of polygenomic infections at different strain proportion ratios. **E**) Kinships of cotransmitted parasites at different strain proportion ratios.
(TIF)

S7 Fig Relatedness of cotransmitted strains under variable oocyst and infected hepatocyte conditions. Violin plots of the relatedness of cotransmitted strains where oocyst and infected hepatocyte counts were drawn from distributions resembling those in real transmission events. A box plot is drawn in the center of each violin plot, where the white dot represents the median of the distribution, the thicker line represent the interquartile range, and the thinner line represents the whiskers of the box plot, up to 1.5 times the interquartile range. The horizontal dotted line represents the value of 0.33.
(TIF)

S8 Fig 3D7 reference allele proportions in polygenomic infections collected from Thiès, Senegal. Representative 3D7 reference allele proportions in the pileups of all sites with a non-uniform read pileup from three COI = 2 polygenomic infections collected from Thiès, Senegal. These samples were previously sequenced and used in [4]. These reference allele proportions reveal a wide range in strain proportions, ranging from 1:1 to 9:1.
(TIF)

S1 Table Observed chiasma events from progeny of lab crossed strains. Table of the average and standard deviation of the number of chiasma events per chromsosome.
(PDF)
