# Supplementary figures and images for: Modeling the genetic relatedness of Plasmodium falciparum parasites following meiotic recombination and cotransmission
Source: PLoS Comput Biol. 2018 Jan 9;14(1):e1005923. doi: 10.1371/journal.pcbi.1005923 (PMC5777656; doi:10.1371/journal.pcbi.1005923)

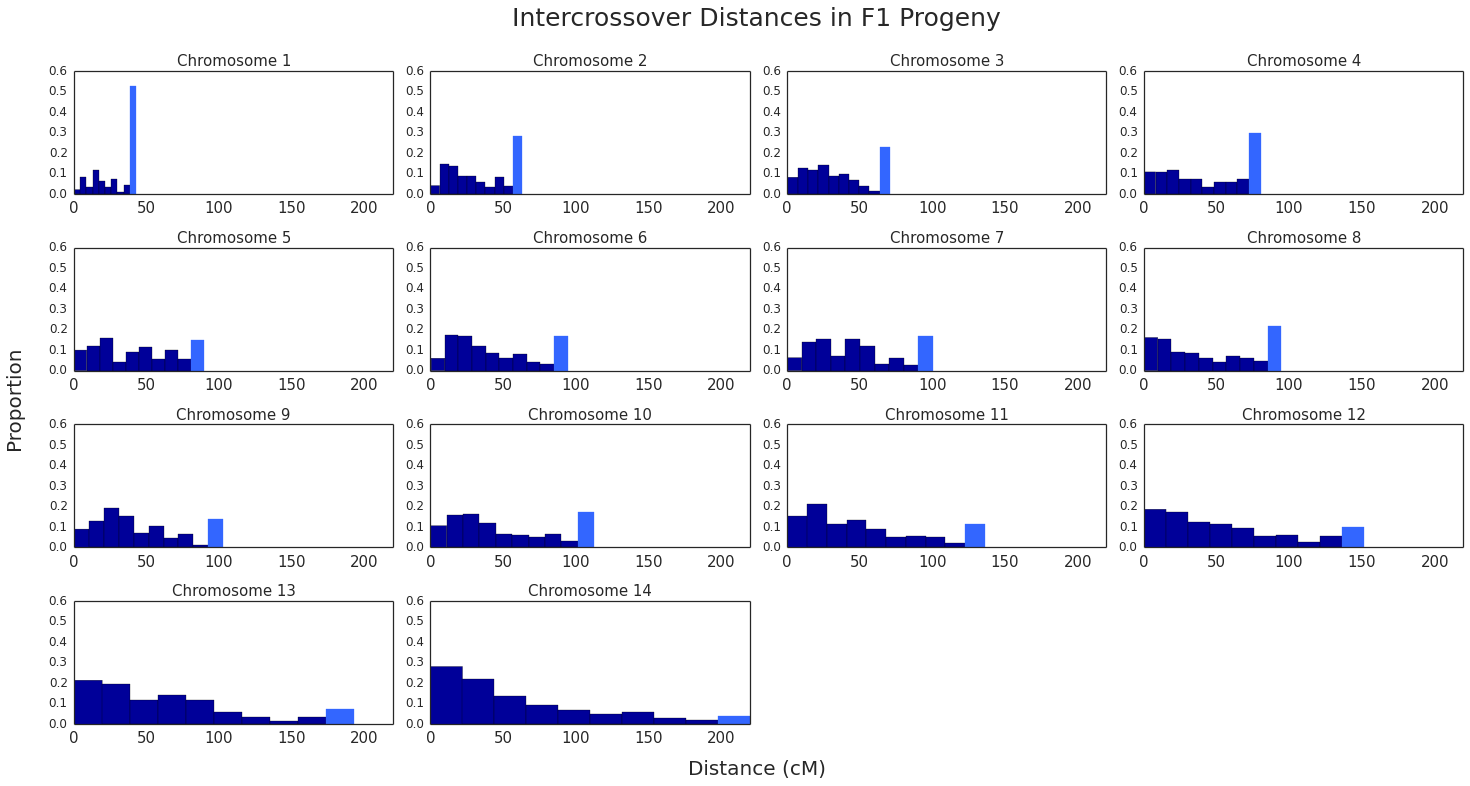

Supplement: S1 Fig — Histograms of the distribution of intercrossover distances (cM) for each chromosome in the P. falciparum genome. Dark blue indicate distances whose boundaries fall within each chromosome and light blue represents distances that span the entire chromosome. (TIF) [file pcbi.1005923.s001.tif]

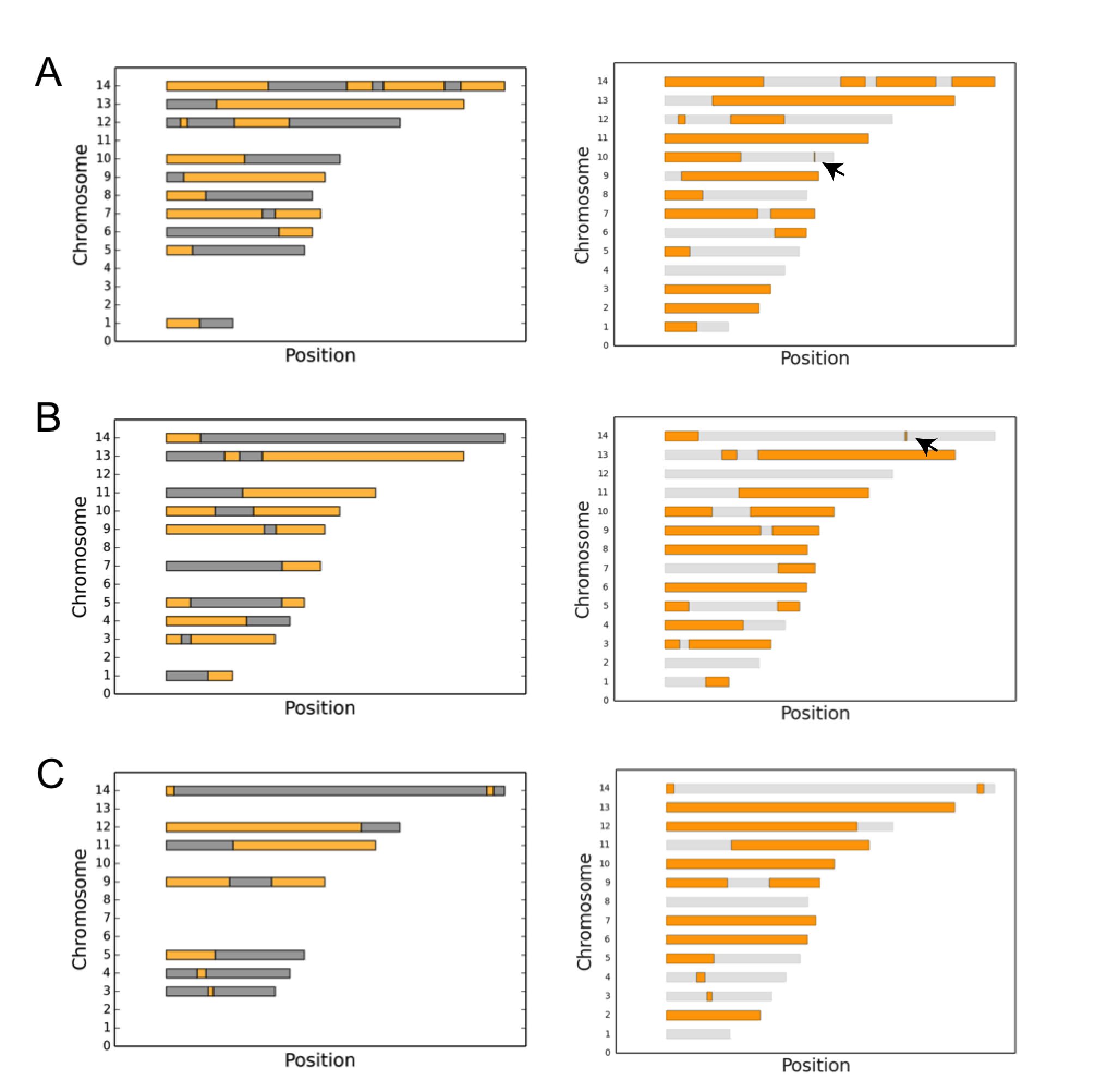

Supplement: S2 Fig — Comparison of the parental inheritance boundaries defined by [16] (left) and our HMM (right) for A) 3D7_ERR019061 (parental) vs C12_ERR019063 (progeny) B) 7G8_ERR027099 (parental) vs AUD_ERR029406 (progeny) and C) DD2_ERR012840 (parental) vs 3BA6_ERR126027 (progeny). For the maps based on the boundaries defined by [16], only the results from chromosomes with evidence of recombination are shown. Orange coloration indicates a section of the genome inherited from 3D7_ERR019061, 7G8_ERR027099, or DD2_ERR012840 while grey sections indicate a section inherited by the other parent in the cross. Our HMM occasionally identified short IBD segments (marked with arrows) not present in the data from [16]. (PNG) [file pcbi.1005923.s002.png]

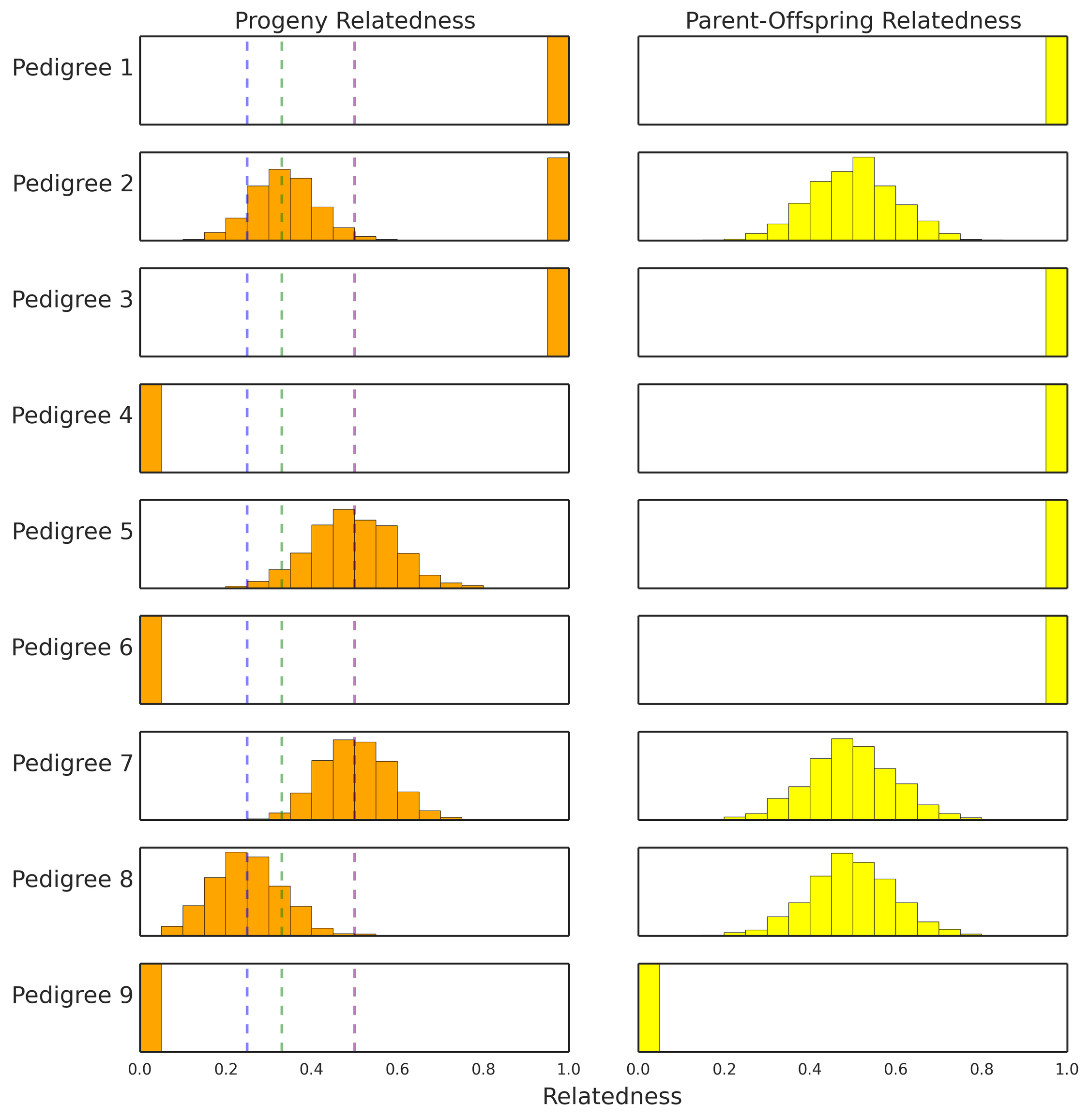

Supplement: S3 Fig — Histograms of the expected relatedness for each pedigree. Orange: Relatedness of between progeny strains. Yellow: relatedness of progeny strains vs one of the parental strains. The blue dotted line represents the expected relatedness of half-siblings (0.25), the green dotted line represents the expected relatedness of unique meiotic siblings (0.33), and the purple dotted line represents the expected relatedness of full-siblings / parent-offspring strains (0.5). (TIF) [file pcbi.1005923.s003.tif]

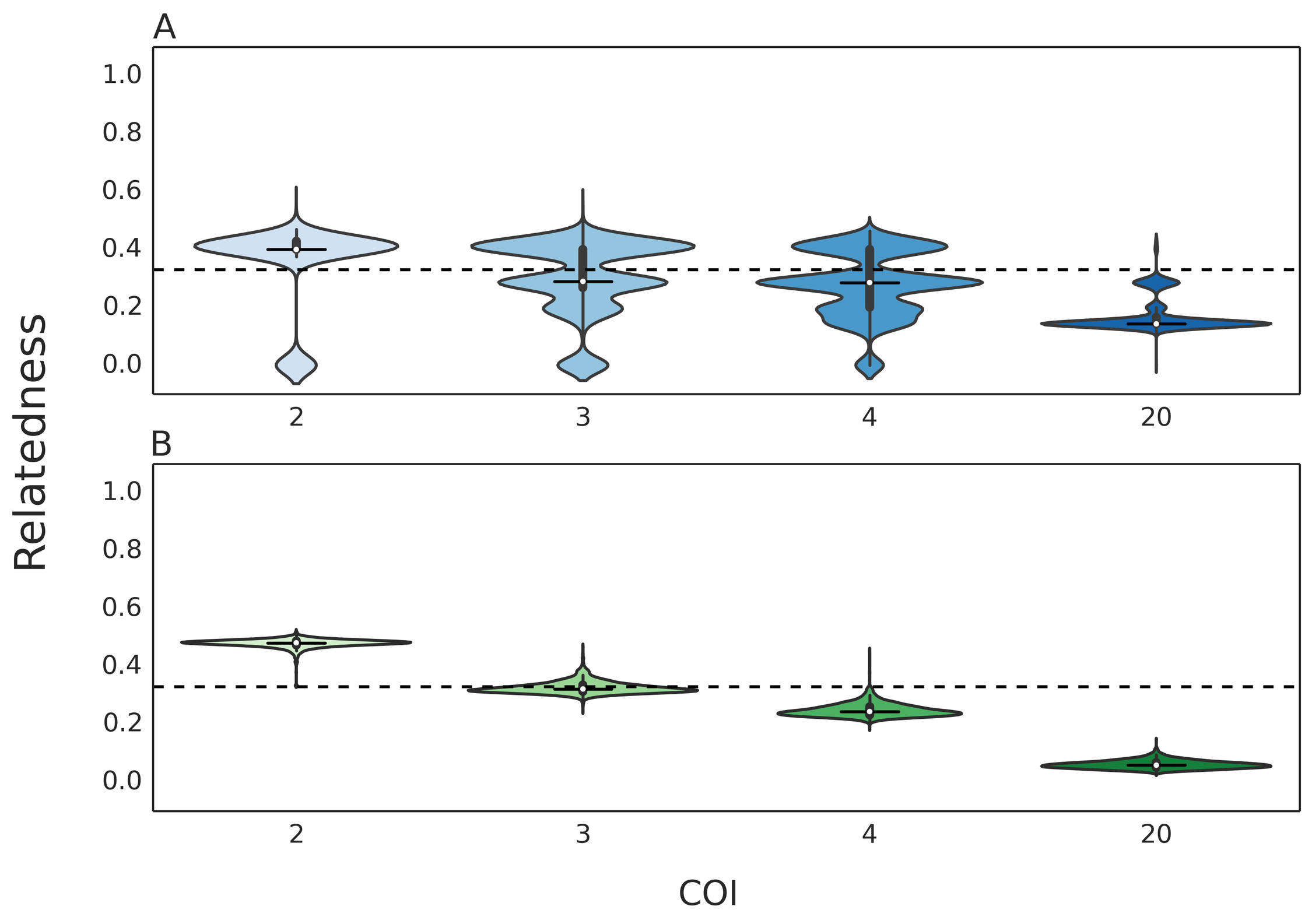

Supplement: S4 Fig — Violin plots of the relatedness of cotransmitted strains in simulations where the infected hepatocyte count was 20 and the oocyst count was 2 (A) or 20 (B). A box plot is drawn in the center of each violin plot, where the white dot represents the median of the distribution, the thicker line represent the interquartile range, and the thinner line represents the whiskers of the box plot, up to 1.5 times the interquartile range. The horizontal dotted line represents the value of 0.33. (TIF) [file pcbi.1005923.s004.tif]

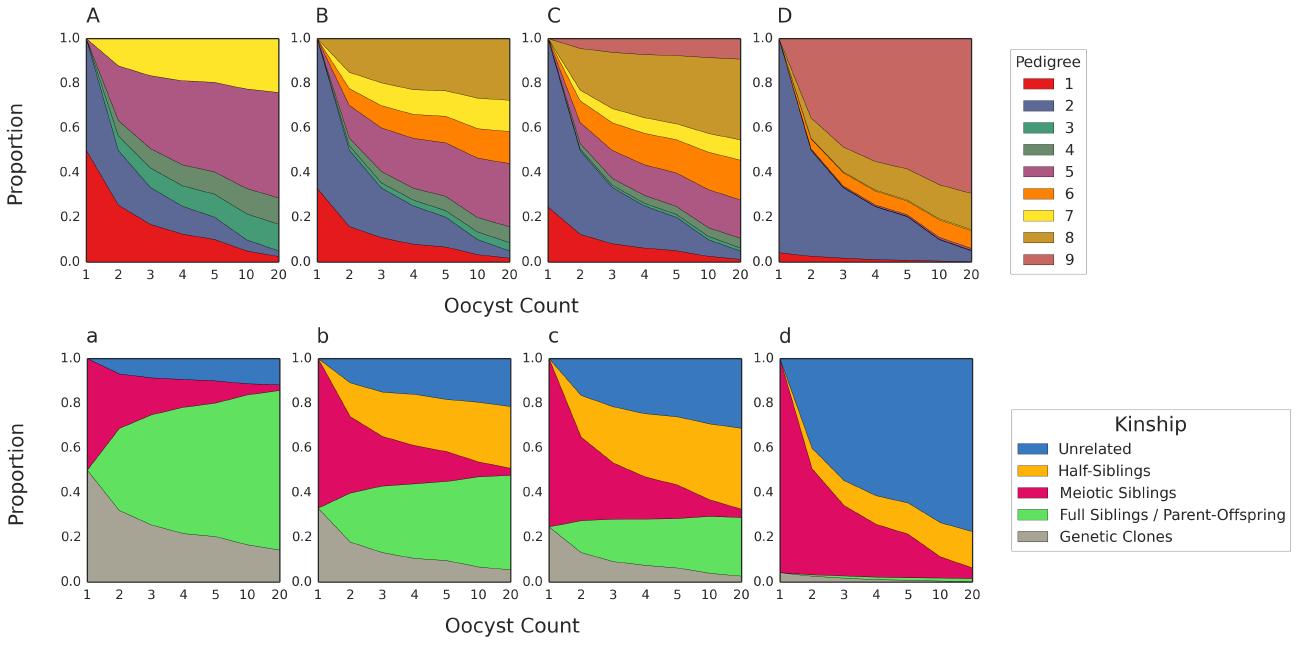

Supplement: S5 Fig — Stacked line charts of the frequencies of different pedigrees (A-D) and kinships (a-d) plotted against oocyst count. Each subplot represents a scenario with a different COI (A/a = 2, B/b = 3, C/c = 4, D/d = 20). Results from simulations where infected hepatocyte count = 20 are shown. Genetic clones are defined as those emerging from oocysts characterized by pedigree 1 and 3; genetically identical meiotic siblings are still classified as meiotic siblings in this graph. (TIF) [file pcbi.1005923.s005.tif]

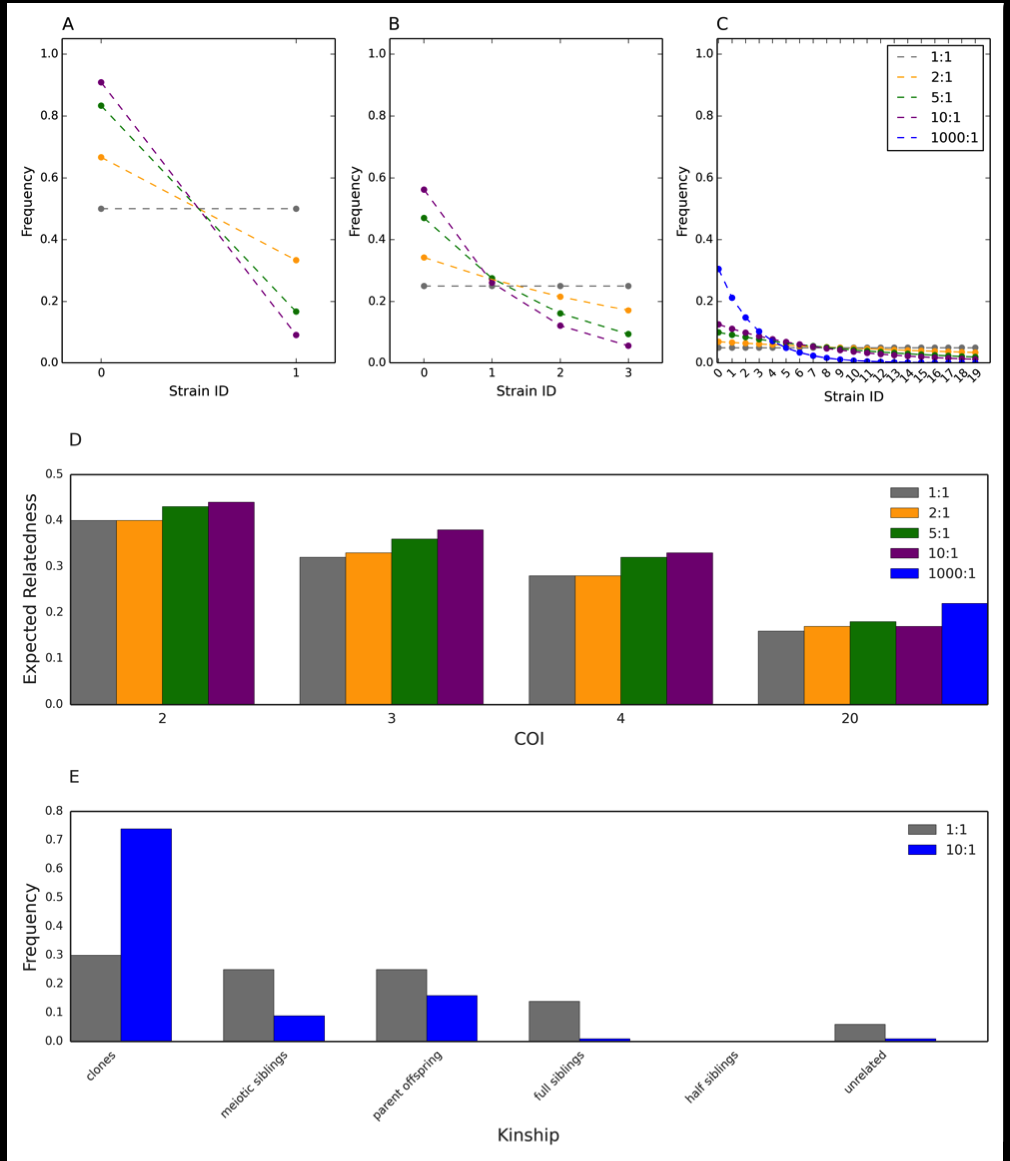

Supplement: S6 Fig — Strain frequencies in COI 2 (A), 4 (B) and 20 (C) infections. We examined strain proportions ranging from 1:1 to 10:1 for all COI infections. We also examined a 1000:1 ratio for COI = 20 infections. Ratios exceeding 10:1 were not examined in the COI = 2 and COI = 4 infections because the minor strains become so infrequent that the infections could be considered lower COI infections. B) Expected relatedness of cotransmitted strains after a single cotransmission event. Only results using oocyst and infected hepatocyte counts of 2 are shown. C) Kinships among transmitted parasites from infections with different strain proportions. Only results using oocyst and infected counts of 2 from a COI = 2 infection are shown. D) Expected relatedness of polygenomic infections at different strain proportion ratios. E) Kinships of cotransmitted parasites at different strain proportion ratios. (TIF) [file pcbi.1005923.s006.tif]

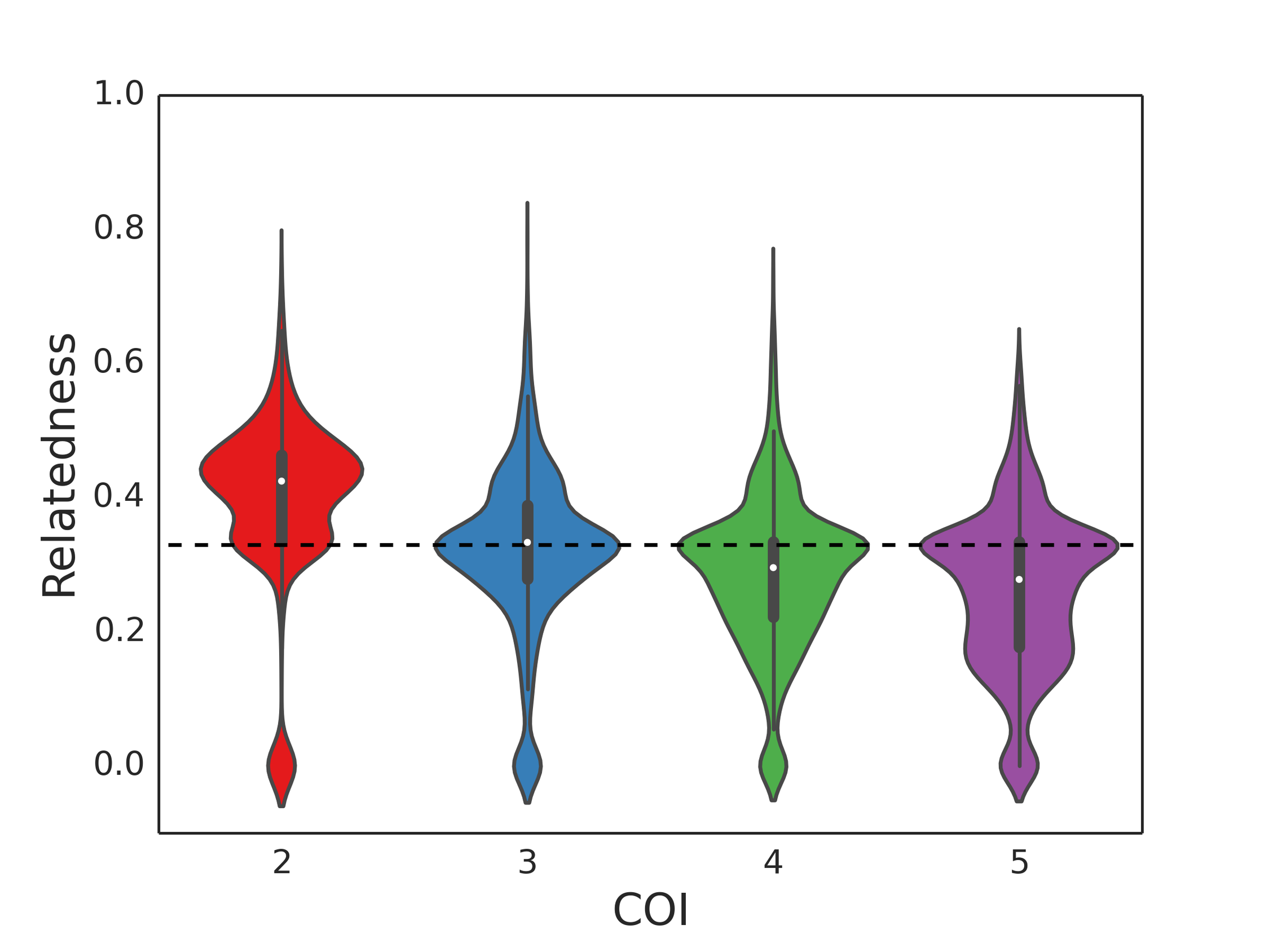

Supplement: S7 Fig — Violin plots of the relatedness of cotransmitted strains where oocyst and infected hepatocyte counts were drawn from distributions resembling those in real transmission events. A box plot is drawn in the center of each violin plot, where the white dot represents the median of the distribution, the thicker line represent the interquartile range, and the thinner line represents the whiskers of the box plot, up to 1.5 times the interquartile range. The horizontal dotted line represents the value of 0.33. (TIF) [file pcbi.1005923.s007.tif]

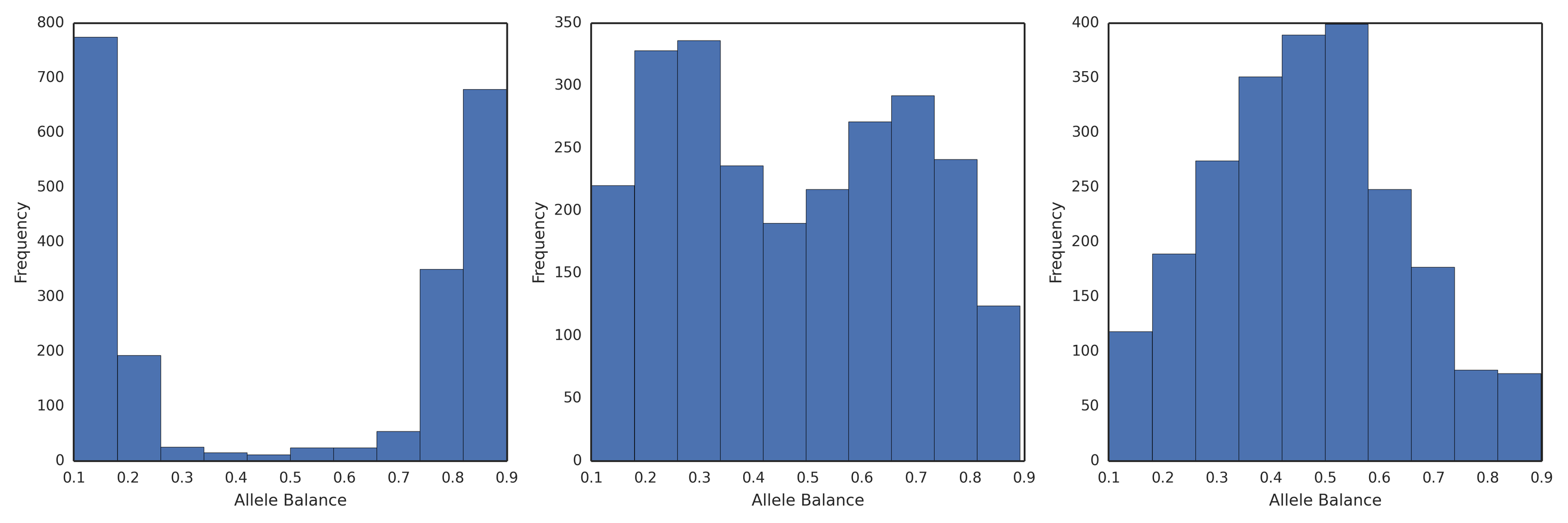

Supplement: S8 Fig — Representative 3D7 reference allele proportions in the pileups of all sites with a non-uniform read pileup from three COI = 2 polygenomic infections collected from Thiès, Senegal. These samples were previously sequenced and used in [4]. These reference allele proportions reveal a wide range in strain proportions, ranging from 1:1 to 9:1. (TIF) [file pcbi.1005923.s008.tif]
