# Supplementary material for: Modeling the genetic relatedness of Plasmodium falciparum parasites following meiotic recombination and cotransmission
Source: PLoS Comput Biol. 2018 Jan 9;14(1):e1005923. doi: 10.1371/journal.pcbi.1005923 (PMC5777656; doi:10.1371/journal.pcbi.1005923)
Supplement: S1 Table — Table of the average and standard deviation of the number of chiasma events per chromsosome. (PDF) [file pcbi.1005923.s009.pdf]

Chromosome	Average Number of Chiasma Events	Standard Deviation
**1**	0.38	0.68
**2**	0.78	0.99
**3**	0.96	1.01
**4**	0.75	0.84
**5**	1.01	0.83
**6**	1.14	1.03
**7**	1.06	1.1
**8**	1.06	1.2
**9**	1.26	1.04
**10**	1.19	1.13
**11**	1.55	1.41
**12**	1.57	1.31
**13**	1.97	1.34
**14**	2.45	1.66
